# Mahanine exerts *in vitro* and *in vivo* antileishmanial activity by modulation of redox homeostasis

**DOI:** 10.1038/s41598-017-03943-y

**Published:** 2017-06-23

**Authors:** Saptarshi Roy, Devawati Dutta, Eswara M. Satyavarapu, Pawan K. Yadav, Chhabinath Mandal, Susanta Kar, Chitra Mandal

**Affiliations:** 1 Cancer Biology and Inflammatory Disorder Division, Council of Scientific and Industrial Research (CSIR)-Indian Institute of Chemical Biology, 4, Raja S.C. Mullick Road, Jadavpur, Kolkata, 700032 India; 2 Division of Parasitology, CSIR-Central Drug Research Institute, Lucknow, 226001 India; 3 National Institute of Pharmaceutical Education and Research, Kolkata, 4, Raja S. C. Mullick Road, Kolkata, 700032 India

## Abstract

Earlier we have established a carbazole alkaloid (mahanine) isolated from an Indian edible medicinal plant as an anticancer agent with minimal effect on normal cells. Here we report for the first time that mahanine-treated drug resistant and sensitive virulent *Leishmania donovani* promastigotes underwent apoptosis through phosphatidylserine externalization, DNA fragmentation and cell cycle arrest. An early induction of reactive oxygen species (ROS) suggests that the mahanine-induced apoptosis was mediated by oxidative stress. Additionally, mahanine-treated *Leishmania*-infected macrophages exhibited anti-amastigote activity by nitric oxide (NO)/ROS generation along with suppression of uncoupling protein 2 and Th1-biased cytokines response through modulating STAT pathway. Moreover, we have demonstrated the interaction of a few antioxidant enzymes present in parasite with mahanine through molecular modeling. Reduced genetic and protein level expression of one such enzyme namely ascorbate peroxidase was also observed in mahanine-treated promastigotes. Furthermore, oral administration of mahanine in acute murine model exhibited almost complete reduction of parasite burden, upregulation of NO/*iNOS*/ROS/IL-12 and T cell proliferation. Taken together, we have established a new function of mahanine as a potent antileishmanial molecule, capable of inducing ROS and exploit antioxidant enzymes in parasite along with modulation of host’s immune response which could be developed as an inexpensive and nontoxic therapeutics either alone or in combination.

## Introduction

*Leishmania* is an obligatory intracellular protozoan parasite responsible for the development of a spectrum of disease manifestation ranging from cutaneous to the more destructive visceral form^1^. Visceral leishmaniasis (VL) is a neglected tropical disease, mainly caused by the species *L. donovani*, which is prevalent in the Indian subcontinent with 40,000 cases registered each year and 147 million people under the risk^2^.

Macrophages are the primary host for the parasite to survive and multiply in the mammalian system. Development of antileishmanial immunity depends on the Th1 type immune response generated by IL-12 secretion by antigen presenting cells (APCs) which in turn induce IFNγ secretion by T cells. This secreted IFNγ further induce macrophages for generation of nitric oxide (NO) and reactive oxygen species (ROS), which are the major antileishmanial defense molecules^3^. Uncoupling protein (UCP) is a mitochondrial membrane transporter which takes part in the regulation of mitochondrial ROS generation in macrophages^4^. *Leishmania* developed several strategies to dodge the host immune response to the establishment of successful infection in the hostile environment. This parasite induces the expression of negative regulatory protein UCP2 in macrophages as well as utilizes their own cascade of antioxidant enzymes like ascorbate peroxidase (APX), glutathione synthetase, tryparedoxin peroxidase for the suppression of ROS generation thereby neutralizing oxidative stress in host for their survival^5–8^.

Due to unavailability of effective vaccines, treatment solely relies on chemotherapy. Although pentavalent antimonials were the mainstream therapy for past 70 years, a large percentage of patients are resistant to this drug. Currently, amphotericin B (conventional deoxycholate or liposomal formulations) has emerged as the first line of treatment. Miltefosine is the only oral drug. However, emerging resistance to miltefosine is particularly worrying. Alongside, most of these synthetic antileishmanial drugs are highly expensive and suffer from various side effects, long treatment regimen and acute toxicity, thus pose a real challenge for the management and elimination program of this poor man’s disease^1, 9^. With such a scenario, it becomes imperative to develop low-cost antileishmanial molecules with minimal toxicity and immunomodulatory activity from the vast Indian natural resources as the armory of antileishmanial drugs are limited. Thus, an ideal antileishmanial molecule should possess the capability to target the parasite as well as to modulate the immune system of the host.

Mahanine, a carbazole alkaloid, was isolated from the leaves of an edible Indian medicinal plant *Murraya koenigii* abundantly available across the country^10^. Earlier work has established mahanine as a potent anticancer molecule against various cancers having different mutations with minimal toxicity towards normal cells both *in vitro* and *in vivo* ^10–17^.

Miltefosine and cisplatin, two powerful anticancer agents, previously have been proved as potent antileishmanial molecules^18^. However, the involvement of mahanine-induced cell death is not known in any parasitic disease. This led us to investigate the potency of mahanine against *L. donovani*. One of the main objectives of this study was to understand the efficacy and molecular mechanism of mahanine-induced apoptosis and targeting the antioxidant enzymes. Another major objective of our study was to investigate the *in vitro* and *in vivo* efficacy of mahanine for inducing effector molecules along with immunomodulation.

Here we provide evidence that mahanine induced antileishmanial activity both in promastigotes and amastigotes. Next, we have confirmed its potential activity in an acute murine model of leishmaniasis for almost complete clearance of the parasites along with upregulation of NO/*iNOS*/ROS/IL-12 and T cell proliferation. Moreover, we have demonstrated mahanine-mediated ROS generation which facilitates modulation of the ROS-generating pathway in *Leishmania*-infected macrophages and induced apoptosis. Furthermore, *in-silico* docking revealed that mahanine can interact with antioxidant enzymes present in *L. donovani*. To the best of our knowledge, this is the first report about the important role of this potent prooxidant molecule in inducing apoptosis both by targeting antioxidant enzyme of the parasite and altered immune regulation in the host for successful clearance of infection.

## Results

### Mahanine exhibited dose-dependent anti-promastigote activity and 7-AAD positivity

**Figure 1:**
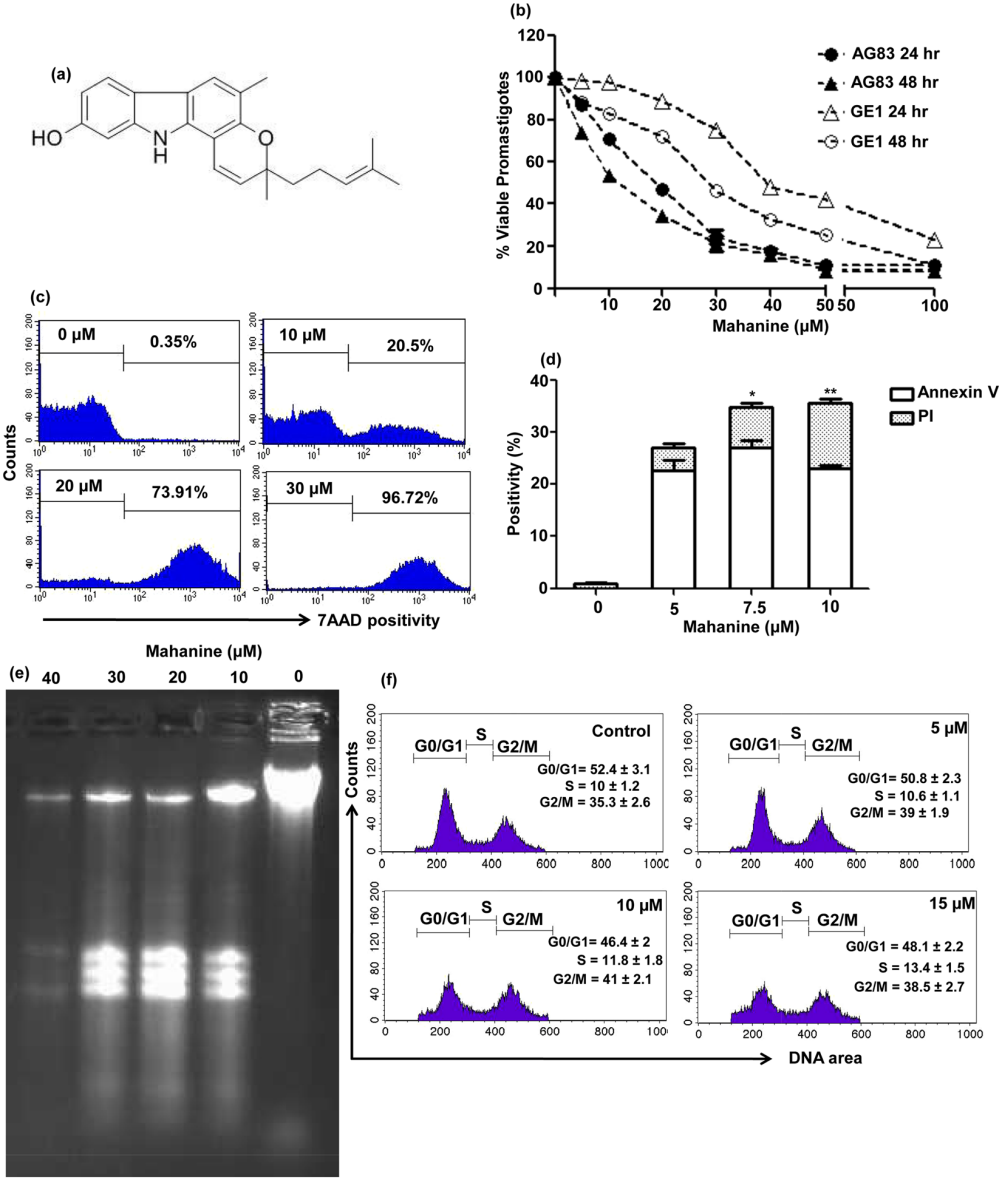
Mahanine triggered apoptotic-like events in virulent promastigotes of *L. donovani*. (**a**) Structure of mahanine. (**b**) Exponentially growing SAG sensitive and resistant strain AG83 and GE1 promastigotes (1 × 10^5^) were plated in each well and incubated with either 1% absolute ethanol (vehicle control) or mahanine (5–100 µM) for 24 and 48 hr at 22 °C. MTS-PMS (5:1) was added and cell viability was measured in ELISA reader at 495 nm. (**c**) Log phase promastigotes (2 × 10^6^/well) were incubated with mahanine (0–30 µM) for 24 hr at 22°C. 7-AAD positivity was quantified by FACS. (**d**) Parasites (2 × 10^6^/well) were either treated with vehicle or mahanine (5.0–10 µM) for 24 hr. Annexin V-PI positive cells were quantified in a flow cytometer. (**e**) Genomic DNA was extracted from mahanine and absolute ethanol (1%)-treated parasites. DNA was separated on 1% agarose gel and visualized with ethidium bromide. (**f**) AG83 promastigote were exposed to either vehicle control or mahanine (5–15 µM) for 24 hr and processed using cell cycle analysis kit. Histograms showed the distribution of DNA content in each stage of cell cycle.

To determine the efficacy, the viability of mahanine-treated virulent drug sensitive and resistant *L. donovani* promastigotes (AG83 and GE1 respectively) was evaluated using the 3-(4,5-dimethylthiazol-2-yl)-5-(3-carboxymethoxyphenyl)-2-(4-sulfophenyl)-2H-tetrazolium inner salt and phenazine methosulfate (MTS-PMS) solution, a modified MTT assay. Reduction in formazan production is due to decrease in mitochondrial activity indicating enhanced cell death. Mahanine (0–50 µM) induced a dose-dependent decrease in cell viability of AG83 promastigotes after 24 hr and 48 hr; the IC_50_ values were 16.7 ± 1.7 µM and 11.5 ± 0.8 µM respectively (Fig. 1b). In a drug resistant GE1 strain, mahanine treatment showed dose-dependent cell death in 24 and 48 hr treatment with IC_50_ values 40.3 ± 2.2 µM and 29.1 ± 1.3 µM respectively (Fig. 1b). Ethanol was used as the vehicle control and displayed no apparent toxicity on the both the parasite strains.

Apoptosis or necrosis resulted in membrane damage and increased positivity of 7-Aminoactinomycin D (7-AAD), a DNA binding dye. Thus increased 7-AAD positivity also indicates the cell death. Mahanine (10 µM) treatment resulted in 20.5% 7-AAD positivity while at 30 µM dose it was 96.7% indicating the complete death of AG83 promastigotes (Fig. 1c).

### Mahanine-mediated alteration of phosphatidylserine and PI positivity

Annexin V has the affinity towards phosphatidylserine, a key molecule in apoptotic signaling. Propidium iodide (PI) is a DNA binding dye. As annexin V positivity is the early event during apoptosis, this assay was performed with the lower doses than IC_50_.

Annexin V positivity was increased to 22.72 ± 1.84% and 27.0 ± 2.06% in mahanine (5.0 µM and 7.5 µM)-treated cells with low PI positivity (4.37 ± 0.91% and 7.78 ± 1.07%) respectively after 24 hr. At the 10 µM dose, PI positivity further increased to 12.66 ± 1.91%, indicating promastigotes are entering into the late apoptotic phase (Fig. 1d). Untreated log phase promastigotes exhibited no binding with annexin V.

### DNA degradation in the mahanine-treated promastigotes

Degradation of nuclear DNA by endogenous nuclease gives the characteristic DNA laddering, which is one of the hallmarks of apoptotic cell death. Genomic DNA isolated from untreated promastigotes exhibited as a tightly packed band near the loading well and barely moved (Fig. 1e). However, DNA isolated from mahanine (10–30 µM)-treated cells were fragmented. No packed and coiled DNA was visible at 40 µM dose.

### Mahanine induced cell cycle arrest at G2/M phase

The percentage of cells in the different phase of the cell cycle was quantified by using PI and a number of bound dye correlates with the DNA content. Mahanine (5.0 and 10 µM)-treated cells exhibited increased accumulation of cells at G2/M phase being 39.0 ± 1.90% and 41.0 ± 2.10% respectively compared to untreated promastigotes (35.3 ± 2.60%), while the number of cells at G0/G1 phase were decreased at 10 µM than control. At a higher dose, cell population decrease in G2/M and it moved to apoptosis. A representative flow diagram of cell cycle arrest along with the percentage of the cell population is shown in Fig. 1f.

### Mahanine induced intracellular ROS in promastigotes

**Figure 2:**
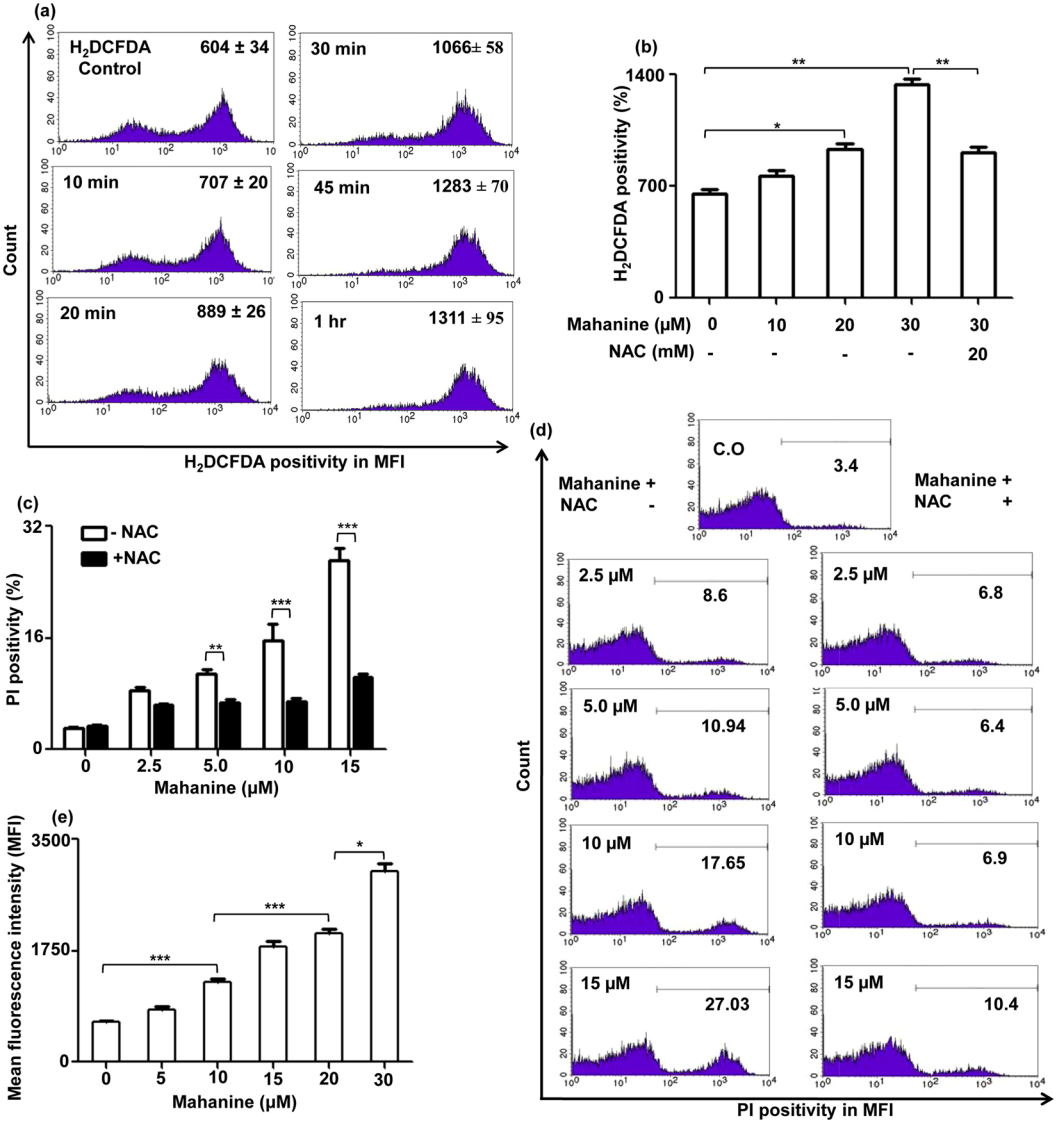
Mahanine-mediated mitochondrial dysfunction, ROS generation, and ROS-dependent cell death in AG83 virulent promastigotes. (**a**) Promastigotes (2 × 10^6^) were preloaded with H_2_DCFDA (50 µM) and plated in a six-well plate. The IC_90_ dose of mahanine (25 µM) was used at different time points (0–1 hr). H_2_DCFDA positivity was measured by FACS. (**b**) Promastigotes were preloaded with H_2_DCFDA and treated with mahanine (0–30 µM) for 45 min. For the highest concentration of mahanine (30 µM) treatment, promastigotes were pretreated with NAC (20 mM). Cells were acquired in FACS and analyzed. (**c**) Promastigotes (2 × 10^6^) were either untreated or treated with NAC (20 mM) and incubated with mahanine (0–15 µM) for 24 hr at 22 °C. Promastigotes were stained with PI and analyzed in FACS. (**d**) A representative image was shown by a histogram. (**e**) Promastigotes were either treated with ethanol (1%) or mahanine (0–30 µM) for 24 hr at 22 °C.Parasites were incubated with JC1 for 30 min, processed and analyzed by FACS.

Mahanine has already been proved as a potent generator of ROS at an early time point in cancer cells^16^. So the efficacy of mahanine in ROS generation was assessed in promastigotes (AG83). Mahanine (25 µM)-treated cells exhibited significantly increased intracellular ROS level within 20 min (MFI being 889 ± 26) which reached to 1288 ± 56 after one hour compared to the basal level (604 ± 34) in untreated promastigote (Fig. 2a).

Based on this result, 45 min timing was selected for ROS generation to optimize the dose. ROS generation was significantly increased MFI being 913 ± 115 and 1237 ± 130 (p ≤ 0.01) when 20 µM and 30 µM of mahanine was used (Fig. 2b) compared to untreated cells (649 ± 40). Pre-treatment of cells with the glutathione precursor N-acetyl cysteine (NAC) successfully inhibited the mahanine (30 µM)-induced ROS level from 1237 ± 119 to 929 ± 59.

### Mahanine-induced ROS contributed to cell death

Next, we were keen to examine the role of this intracellular stress by mahanine-induced ROS in the cell death of promastigotes. Accordingly, mahanine-mediated cell death of parasites was assessed by PI staining in the presence and absence of NAC, the ROS scavenger, after 24 hr.

Mahanine (10 µM and 15 µM) induced 18.85 ± 1.39% and 26.06 ± 2.25% PI positivity in promastigotes which showed a significant (p ≤ 0.001) reduction (7.15 ± 1.12% and 10.97 ± 1.44% respectively) of PI positivity in presence of NAC (Fig. 2c). Thus NAC rescued the effects of the mahanine-induced cell death to a level of ~ 72% suggesting an initial important role of ROS in the mahanine-induced apoptosis of these virulent parasites (Fig. 2d).

### Mitochondrial membrane depolarization in mahanine-treated promastigotes

The enhanced production of ROS increased the option that mitochondrial function may be changed. Accordingly, we measured the membrane potential in mitochondria using 5,5′,6,6′-tetrachloro-1,1′,3,3′-tetraethylbenzimi-dazolylcarbocyanine iodide (JC1). The enhanced green fluorescence indicated the loss of mitochondrial membrane potential. Mahanine-treated promastigotes exhibited dose-dependent upregulation of the green fluorescence. It increased the green fluorescence (MFI 1258 ± 119) in promastigotes compared to untreated cells (MFI 641 ± 42) after 24 hr treatment at 10 µM dose. At the higher doses (25 and 30 µM), the MFI of green fluorescence was further increased to 2478 ± 109 and 3000 ± 206 respectively (Fig. 2e).

### Mahanine reduced the intracellular amastigote number in infected macrophages

Promastigotes are readily phagocytosed by the macrophages into the phagolysosomal vacuole and converted into the nonmotile amastigotes form which is replicative inside the mammalian cells. Accordingly, we were keen to examine whether mahanine also could kill these intracellular infectious form of the parasite.

**Figure 3:**
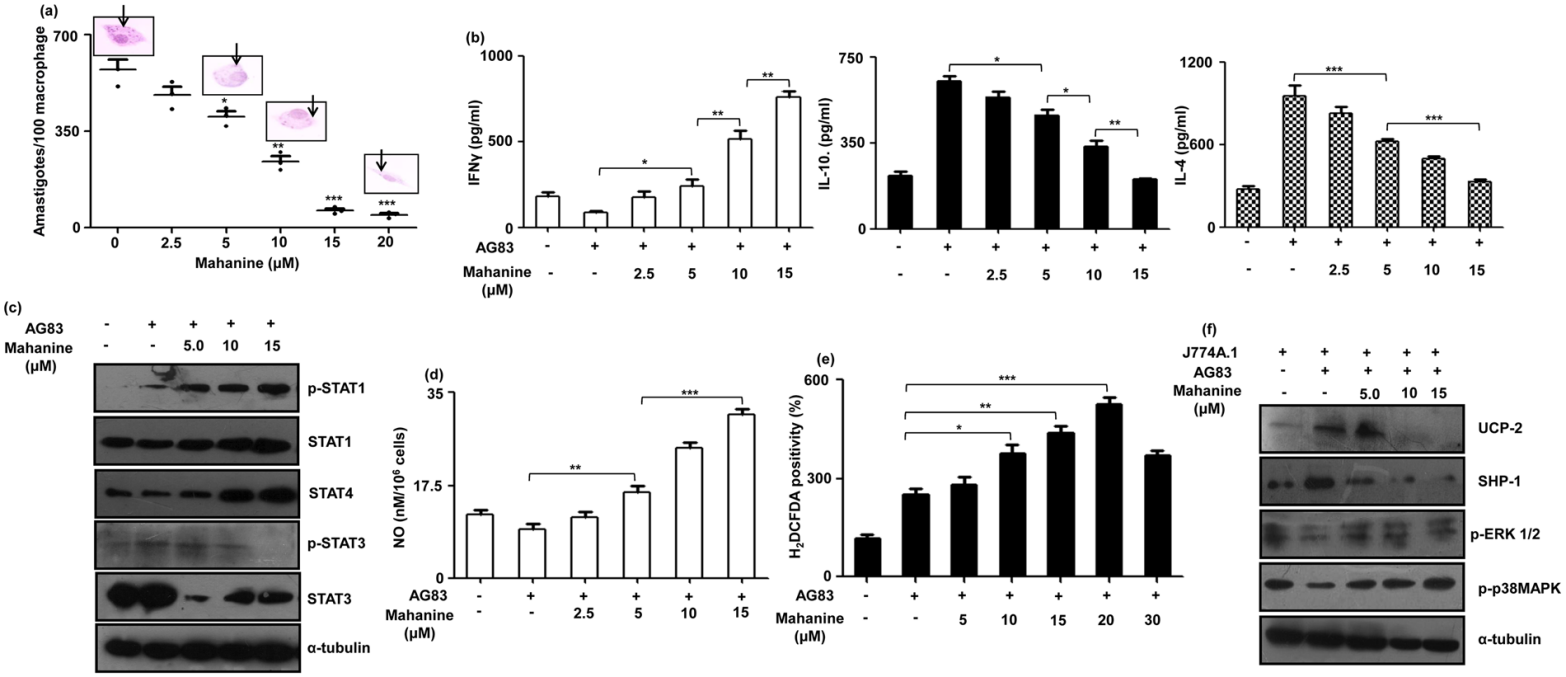
*In vitro* efficacy of mahanine. (**a**) Murine macrophage cell line J774A.1 (2 × 10^4^) grown in 22mm^2^ glass cover slip were infected with stationary phase virulent AG83 promastigotes (1: 10) for 4 hr. Unbound parasites were removed and infection was allowed for another 20 hr. Infected cells were treated with mahanine (0–20 µM) for 24 hr. Macrophages were fixed and stained with Giemsa. Intracellular parasites were counted in a light microscope. The values represented as a mean of three independent experiments. A representative image was given as micro photo above the mean value. (**b**) Macrophages (J774A.1, 1 × 10^6^/well) were infected with stationary phase *L. donovani* (AG83) promastigotes similarly as stated above. The secreted cytokines (IL-4, IL-10, and IFNγ) in the culture supernatants were measured by respective ELISA kit as described in material and methods. (**c**) J774A.1 cells (1 × 10^6^/well) were infected and treated similarly as stated above. The cell lysate was prepared; proteins were quantified, separated in SDS-PAGE. They were transferred to nitrocellulose membrane and incubated overnight with anti-STAT1, p-STAT1, STAT-4, STAT3, p-STAT3 and α-tubulin antibodies. The blots were incubated with respective secondary antibody and developed by ECL kit. α-tubulin was used as a control. (**d**) J774A.1 cells (1 × 10^6^/well) were either uninfected or infected with promastigotes and treated with mahanine (0–15 µM) for 24 hr similarly as stated earlier. The NO secretion was measured in the culture supernatant by Griess reaction. (**e**) J774A.1 cells (1 × 10^6^/well) were left uninfected or infected with promastigotes at 1:10 ratio for 4 hr. Cells were incubated with mahanine (0–30 µM) for 1 hr and Intracellular ROS production was measured after H_2_DCFDA staining by FACS. Data was analyzed by CellQuestPro software. (**e**) J774A.1 cells were infected with AG83 promastigotes similarly as stated above and cell lysate was prepared. The lysate was run in gel, incubated with specific anti-SHP-1, anti-UCP 2, anti-p-p3MAPK, anti-p38MAPK, anti-p-ERK1/2 antibodies and developed similarly. α-tubulin was used as loading control.

Mahanine reduced the amastigotes number in infected macrophages in a dose-dependent manner. A significant decrease was observed when mahanine was used at 5.0 µM, 10 µM and 20 µM doses; values being 402 ± 33 (p ≤ 0.05), 239 ± 32 (p ≤ 0.01) and 46 ± 11 (p ≤ 0.001) respectively compared to 573 ± 61 in untreated infected macrophages (Fig. 3a). The IC_50_ value of mahanine against amastigote was of 8.94 ± 1.20 µM. A representative image of Giemsa staining of infected macrophage in the index of the graph also showed a very low number of amastigotes as pink dots. However, under the similar condition, the viability of macrophages was ≥93% even when highest dose of mahanine was used.

### Induction of Th1 dominant cytokines in mahanine-treated infected macrophages

*Leishmania* infection is associated with the Th2-dominated cytokine response which causes the immune suppressive condition in the host. IL-10 and IL-4 are two major immune suppressive cytokines associated with VL. IFNγ is the Th1 cytokine which gives protection in VL and generally down regulated during infection. To examine whether the mahanine-induced antileishmanial activity was also due to alteration of host’s cytokine response, we investigated the cytokine production inside the infected macrophages before and after treatment with mahanine.

IFNγ secretion dose-dependently increased in mahanine-treated infected macrophages compared to untreated cells (Fig. 3b). At the 5.0 µM, 10 µM and 15 µM doses, the IFNγ secretion increased significantly to 239 ± 65 pg/ml, 513 ± 83 and 756 ± 61 pg/ml respectively compared to untreated cells (87 ± 17). In contrast, IL-10 secretion reduced to 462 ± 38 pg/ml, 333 ± 51 and 201 ± 12 pg/ml at 5.0,10 and 15 µM of mahanine compared to 601 ± 40 pg/ml during infection. The basal level of IL-10 in uninfected cells was only 215 ± 33 pg/ml. Similarly mahanine (5.0 and 15 µM) also decreased the level of another Th2 cytokine (IL-4) significantly to 623 ± 35 pg/ml (p ≤ 0.001) and 330 ± 29 pg/ml (p ≤ 0.001) in infected macrophages compared to 953 ± 132 pg/ml in untreated cells.

### Mahanine exhibited modulation of STAT signaling pathway

Mahanine shifted Th1-biased response by modulating the Th1/Th2 cytokine balance in the infected macrophages. This led us to investigate the cytokine signaling STAT pathway (Fig. 3c). During *Leishmania* infection, there was a reduced expression of p-STAT1, a well-known transcription factor of IFNγ. However, mahanine exhibited a dose-dependent increase of p-STAT1 level in the *Leishmania*-infected macrophages, while total STAT1 remained unaffected. Similarly STAT4, the transcription factor of another Th1 cytokine (IL-12), also showed dose-dependent upregulation in mahanine-treated infected cells compared to untreated infected cells. In contrast, mahanine down regulated the expression of STAT3 and p-STAT3, a transcription factor of Th2 cytokine (IL-4) in dose-dependent manner.

### Enhanced nitric oxide (NO) production in mahanine-treated infected macrophages

To further elucidate whether mahanine induces host’s immune alteration in the infected macrophages for its antileishmanial effect, the production of an effector molecule NO was assessed. In general, NO content was reduced (9.5 ± 1.50 nM) in infected macrophages compared to uninfected cells (12.4 ± 0.78 nM). However, mahanine (5.0 µM)-treated infected macrophages exhibited enhanced NO level (16.86 ± 0.61 nM, p ≤ 0.01) in the culture supernatant. A higher dose of mahanine (10 and 15 µM) further increased its level to 24.53 ± 1.98 and 31.36 ± 1.36 nM respectively (p ≤ 0.001) (Fig. 3d).

### Mahanine dose-dependently upregulated ROS in the infected macrophages

ROS is another key molecule involved in antileishmanial defence. Thus any prooxidant agent having the capability to modulate the macrophages for ROS generation can be an effective antileishmanial agent. Promastigote-infected macrophages in general enhanced its ROS level compared to uninfected cells, MFI being 205 ± 33 *vs*.117 ± 12 respectively. However, mahanine (10 µM)-treated infected macrophages further upregulated ROS level (342 ± 59, p ≤ 0.05), which subsequently augmented to 400 ± 37 and 459 ± 62 (p ≤ 0.01) when doses were increased to 15 and 20 µM. At the highest dose (30 µM), there was a drop in the ROS production, indicating 20 µM is the optimum concentration for ROS production (Fig. 3e).

### Mahanine modulated ROS-generating pathway in the infected macrophages

We have already established that mahanine induced ROS in the infected macrophages. As UCP2 is negatively regulated by *Leishmania* to inhibit mitochondrial ROS production, we have further investigated whether mahanine can inhibit this molecule.

Indeed we observed down regulation of both UCP-2 and SHP-1 in mahanine-treated infected cells in a dose-dependent manner compared to untreated infected cells. Another two signaling molecules (p38MAPK and p-ERK1/2) are generally targeted by SHP-1. Accordingly, we have also monitored their status in mahanine-treated cells. We demonstrated upregulation of both p-p38MAPK and p-ERK1/2 compared to untreated infected cells indicating probability to induce the Th1 response (Fig. 3f).

### Molecular modeling studies of mahanine with antioxidant enzymes

Since *Leishmania* encounter various oxidative stresses during its life cycle, its defense arsenal is enriched with many antioxidant enzymes^8^. As mahanine induces ROS and ROS-mediated cell death both in virulent promastigotes and amastigotes, it was worthwhile to test whether it can target any of these anti-oxidant enzymes of the parasite. As a preliminary study, we performed molecular docking between mahanine and six major antioxidant enzymes of *L. donovani* to examine their affinity and pose of binding (Table 1).

**Table1:** Template identification of six antioxidant enzymes of *L. donovani* on the basis of sequence similarity and e-value.

Antioxidant enzymes	Template (from PDB)	Similarity	e-value
Ascorbate peroxidase	3RIV	96%	0.0
Tryparedoxin peroxidase (cytoplasmic)	3TUE	90%	7e-136
Tryparedoxin peroxidase (mitochondria)	4KB3	89%	2e-127
Superoxide dismutase (FESODA)	4F2N	93%	2e-161
Superoxide dismutase (FESODB1)	3ESF	70%	2e-101
Hsp83	3HJC	96%	0.0

**Figure 4:**
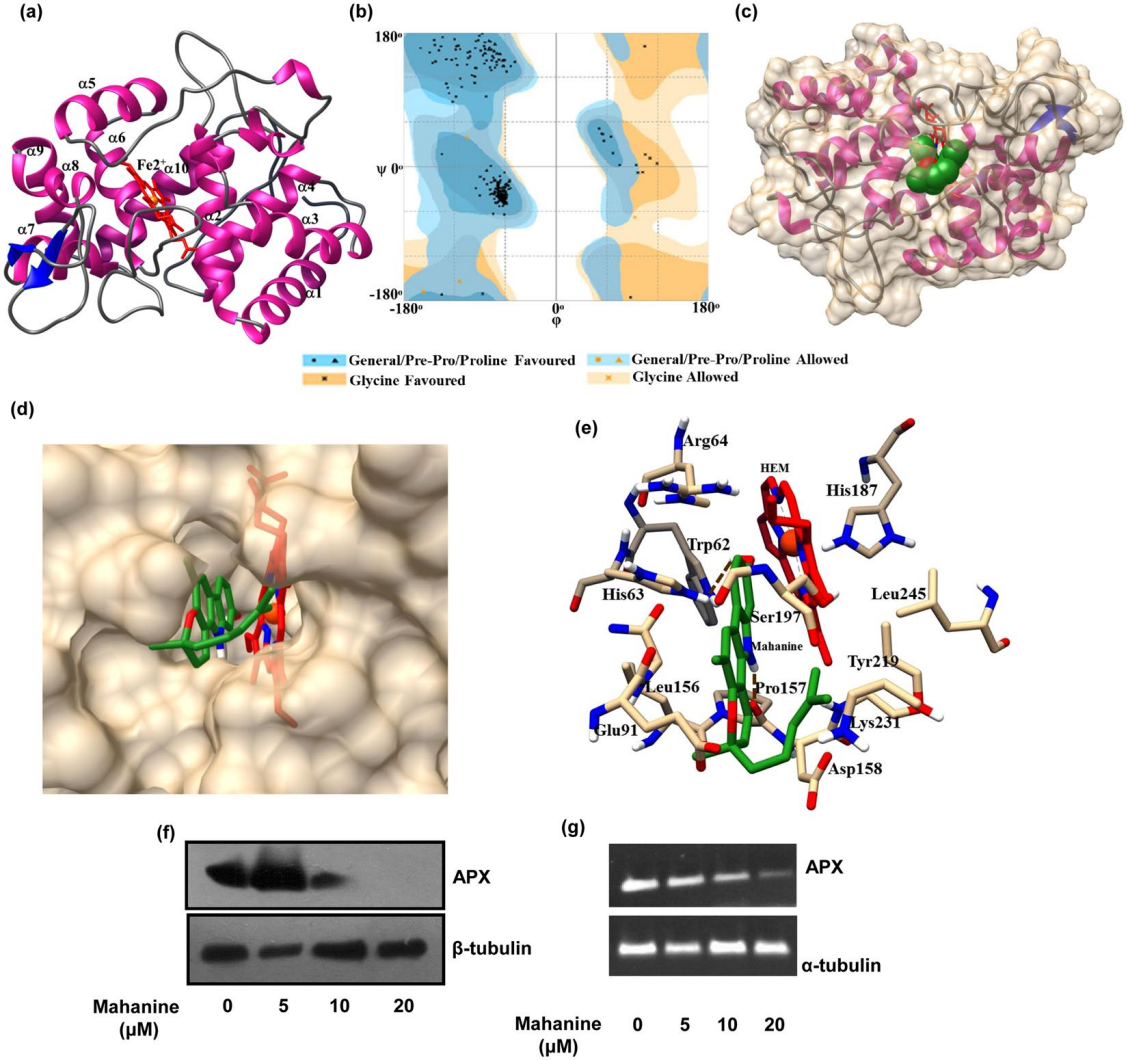
Molecular modeling, genetic and protein level expression profile of ascorbate peroxidase (APX) of *L. donovani* with mahanine. (**a**) Modeled structure of *L. donovani* APX with bound HEM group in the active site. The structure is displayed in ribbon form where colours are represented as (sheets = dark blue, helices = magenta and loop = grey). HEM moiety is represented in sticks in red color and Fe^2+^ in orange. (**b**) Ramachandran plot of the modeled structure. (**c**) Docked complex of mahanine with ascorbate peroxidase. The ligand is represented in sphere form where C = green, O = red, N = blue and H = white. The protein is represented both as a partial Connolly surface in tan color and ribbon form with helices (magenta) and sheets (dark blue). (**d**) Close view of mahanine bound in the active site of the enzyme; mahanine is in close proximity to the HEM. Mahanine and HEM are represented in stick model. (**e**) Amino acid residues of ascorbate peroxidase interacting with mahanine involving H-bond and van der Waals interactions. Both residues and the ligand represented as stick model where colors are highlighted as [carbon (APX) = tan, carbon (mahanine) = green, carbon (HEM) = red, oxygen = red, nitrogen = dark blue, Fe^2+^  = orange]. (**f**) The cell lysate was prepared from mahanine (0–20 µM)-treated log phase promastigotes (2 × 10^7^) for 24 hr and run in SDS-PAGE (12%). The blot was incubated with antibody against ascorbate peroxidase and β-tubulin and developed similarly. (**g**) Promastigotes were treated similarly as stated above and total RNA was isolated. cDNA was prepared, PCR performed using a specific primer of APX and α-tubulin and run in a gel. The image was visualized and photographed in Bio-Rad Gel Documentation system.

Accordingly, three-dimensional structures of all six antioxidant enzymes of *L. donovani* were built using homology modeling, based on the identified templates described in Materials and methods section (Table 1, Fig. 4a, Supporting information Fig. S1A–E). The energy-optimized modeled structures of these antioxidant enzymes showed that the geometry of the main chain was very well favoured in the Ramachandran plot (Fig. 4b, Supporting information Fig. S1A’–E’).

The modeled structure of APX consists of α/β motifs and has RMSD value of 0.10 Å on structural superposition with the template, 3RIV (Fig. 4a, Table 2). Cytoplasmic and mitochondrial tryparedoxin peroxidase comprised a typical thioredoxin fold that contains a central β-sheet and surrounded by α-helices (Supporting information Fig. S1A,B). However, superoxide dismutases and HSP83 consist of mainly α-helices, with some β-sheets in between (Supporting information Fig. S1C–E). Structural validation of all these six enzymes showed that Φ and Ψ backbone torsion angles of most of the non-glycine residues were within the most favoured area, with a few residues in additionally allowed regions. Either one or none of the residues (in some cases) was present as outliers (Table 2, Fig. 4b, Supporting information Fig. S1A′-E′). Structural superposition with the template also showed small RMSD values (less than 1.0 Å). Moreover, the verify3D program which describes the overall quality factor and compatibility of an atomic model (3D) with amino acid sequence (1D) for the modeled protein, showed that more than 80% of the amino acid residues had an averaged 3D-1D score>=0.2, thus satisfying the standard criteria. The overall geometric environment profile calculation of the model determined by the ERRAT program has a good score (Table 2).

**Table 2:** Structure validation of the modeled structure of six antioxidant enzymes of *L. donovani* using different validation programs.

Antioxidant enzymes	Favoured region (%)	Allowed region (%)	Outliers (%)	RMSD with template (Å)	Verify3D program	Errat program (%)
Ascorbate peroxidase	99.2	0.8	Nil	0.10	90.98	96.51
Tryparedoxin peroxidase (cytoplasmic)	98.5	1	1	0.41	96.46	80.0
Tryparedoxin peroxidase (mitochondria)	98.2	1.8	Nil	0.32	80	71.43
Superoxide dismutase (FESODA)	98.1	1.9	Nil	0.12	89	80.0
Superoxide dismutase (FESODB1)	97.9	1.6	1	1.00	100	70.05
Hsp83	98.5	1.2	1	0.33	89	88.0

Molecular docking studies showed that the APX binds to mahanine with higher affinity. However, there are other antioxidant enzymes whose binding affinities are close to that of APX (Table 3, Fig. 4c, Supporting information Fig. S2A–E). This can be attributed to the multi-target nature of mahanine. The location of binding of mahanine is close to HEM group of APX and interacts with a few amino acid residues through non-covalent weak interactions (Fig. 4d,e).

**Table 3:** Free Energy Values of docked complexes of mahanine with antioxidant enzymes of *L. donovani*.

Antioxidant enzymes	Binding energy (ΔG_bind_)Kcal/mol
Ascorbate peroxidase	−7.77
Tryparedoxin peroxidase (cytoplasmic)	−6.58
Tryparedoxin peroxidase (mitochondria)	−6.94
Superoxide dismutase (FESODA)	−5.76
Superoxide dismutase (FESODB1)	−5.85
HSP83	−6.99

Based on these observations, we have selected one such enzyme namely APX for our subsequent study. Accordingly, we monitored the level of APX in mahanine-treated promastigotes after 24 h (Fig. 4f). Mahanine-treated cells exhibited a dose-dependent reduction in the protein level at 10 µM compared to untreated cells. At the highest dose (20 µM), APX expression was completely absent, indicating the total suppression of this protein by mahanine treatment. β-tubulin was used as loading control. Furthermore, mahanine-treated virulent promastigotes also showed reduced genetic expression of APX level in a dose-dependent manner (Fig. 4g). The loading control α-tubulin level remains unchanged before and after mahanine treatment.

### Mahanine exhibited reduced intracellular parasite burden in infected mouse model *via* induction of NO/*iNOS*/ROS/IL-12 and T cell proliferation

So far we have successfully demonstrated that mahanine exerts strong cytotoxicity and induces apoptosis through enhancing the intracellular ROS level both in promastigotes and amastigotes in infected macrophages and leading to decrease the level of antioxidant enzymes. Next, we investigated whether mahanine is capable of clearing intracellular parasite load in infected mice, a well-established acute model to control *Leishmania* infection. Here we have validated the efficacy of mahanine *in vivo* which corroborated the *in vitro* results.

**Figure 5:**
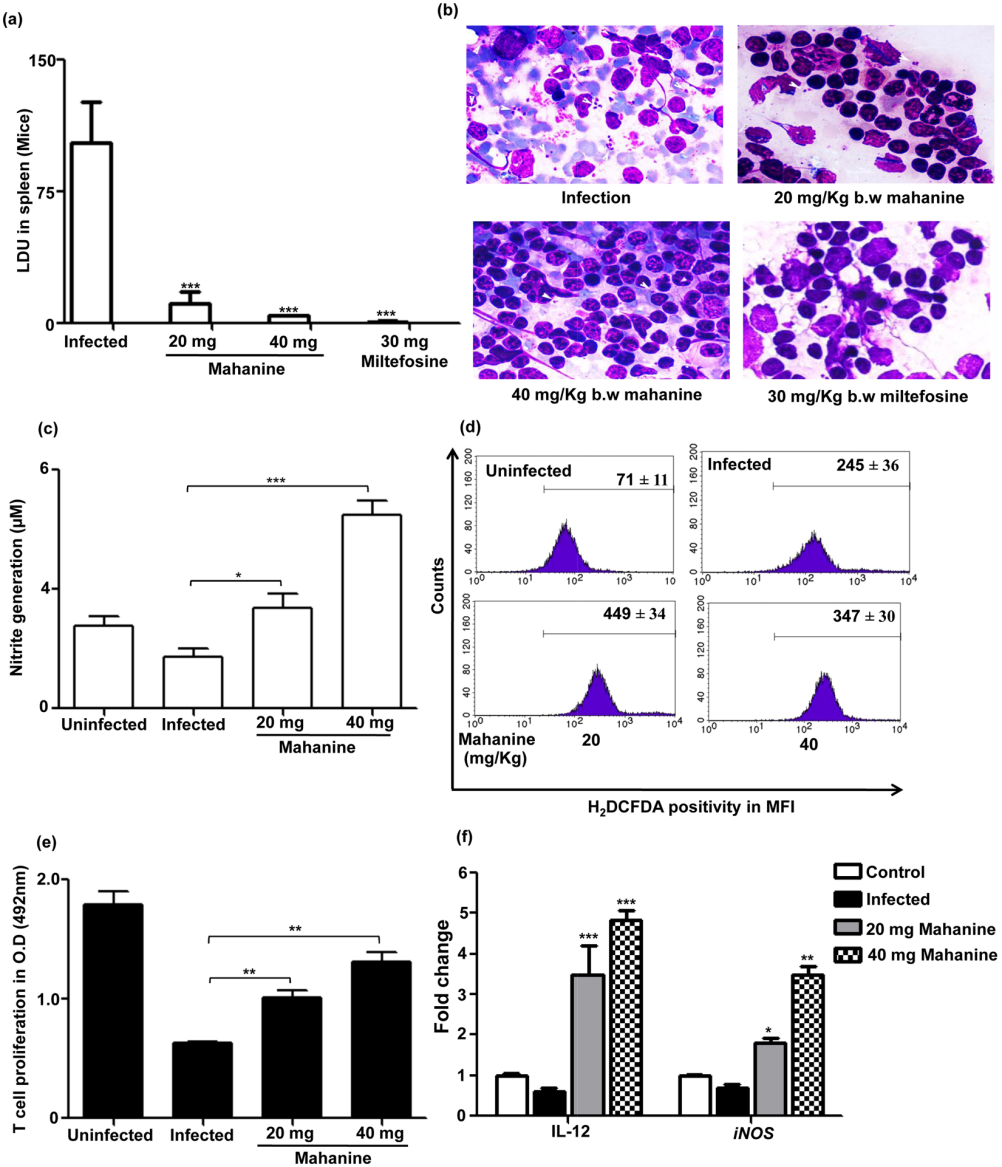
*In vivo* efficacy of mahanine in mice. (**a**) Female Balb/c mice were infected with stationary phase virulent AG83 promastigotes. Infection was established for 15 days and fed with vehicle control (DMSO) or mahanine or miltefosine for five consecutive days. Mice were sacrificed after four-day post feeding and splenic parasite burden was measured by stamp-smear method after Giemsa staining. (**b**) Giemsa-stained infected and treated splenic smears bearing amastigotes as indicated by white arrow (100 X under oil immersion lens). The slides were viewed and the image captured on an inverted bright-field microscope (IX73 inverted microscope; Olympus). (**c**) Splenocytes isolated from infected untreated and treated groups of mice were incubated for 3 days in presence of SLA and supernatant was collected. Nitrite was quantified by Griess reaction. (**d**) Splenocytes isolated from infected and treated groups were plated (1 × 10^6^/ml) in a six-well plate and incubated for 48 hr in presence of SLA. ROS generation was measured by adding H_2_DCFDA in FACS. (**e**) Splenocytes from control and fed mice were plated in a 96 well plate and incubated for 3 days in presence of SLA. Cell proliferation was measured by MTT. (**f**) Total RNA was isolated from infected untreated and treated mice splenocytes and cDNA were prepared. cDNA was amplified by real-time PCR by using specific primers from IL-12 and *iNOS* and represented as fold changes compared to control. HGPRT was used for normalization.

Initially, infection was established in Balb/c mice with virulent AG83 promastigotes and subsequently fed these infected animals with 20 and 40 mg/kg b.w/day of mahanine for 5 consecutive days. Animals were sacrificed 4 days after the last feeding and examined. Fed animals were healthy and no change in body weight was observed. At 20 mg/kg b.w /day dose of mahanine there was 89.1 ± 4.1% reductions in parasite burden in spleen and this decline was almost complete (96.2 ± 0.3%) at 40 mg/kg b.w/day dose even after four days post treatment (Fig. 5a). Standard drug miltefosine also showed complete clearance (~99%) of parasite burden from the infected spleen. A representative image of infected and treated (mahanine and miltefosine) mice splenic tissue is shown in the Fig. 5b.

Mahanine induced modulation of effector function in the infected murine model was tested to further establish the role of mahanine in effector molecule generation. Accordingly, we checked the efficacy of mahanine in NO generation in the culture supernatant of these murine splenocytes isolated from infected untreated and treated groups. Splenocytes in the 20 and 40 mg/kg b.w/day treated mice showed 2.53 ± 0.60 µM and 4.861 ± 0.88 µM nitrite level compared to corresponding infected (1.31 ± 0.36) and uninfected control (Fig. 5c). The efficacy of mahanine in ROS generation in murine splenocytes isolated from infected untreated and treated groups was evaluated. We observed upregulation of ROS level to 449 ± 61 and 347 ± 52 MFI units compared to those isolated from infected mice which showed the basal level of ROS (Fig. 5d).

*Leishmania* infection impaired the T cell proliferation and therefore any ideal antileishmanial molecule should promote lymphocyte proliferation to reduce the parasite burden. Almost complete remission of parasite burden and induction of microbicidal molecules interested us to study the mahanine-induced cell-mediated immunity by measuring T cell proliferation. As expected, infected murine splenocyte showed 65% reduction of T cell proliferation than uninfected control. However, mahanine-treated mice exhibited 37% and 52% fold increased T cell proliferation than infected splenocytes (Fig. 5e).

We observed enhanced Th1 type response in mahanine treated-infected macrophages *in vitro* which is necessary to resolve the *Leishmania* infection. As IL-12 is the key cytokine that induces Th1 cell proliferation, we tested the efficacy of mahanine in murine splenocytes by real time PCR. We found mahanine-fed mice resulted in 3.49 and 4.84 fold increase of IL-12 in mRNA level at both the doses as compared to only 0.61 fold in the infected murine splenocytes indicating the boost in cell-mediated immunity (Fig. 5f). As IL-12 production ultimately leads to the enhancement of effector function, we have tested the mRNA expression level of NO synthase gene, *iNOS*. Consistent with our NO generation data, the *iNOS* mRNA were significantly upregulated (1.80 and 3.48 fold) after 20 and 40 mg/kgb.w/day mahanine treatment compared to infected control (0.68 fold) (Fig. 5f).

## Discussions

Visceral leishmaniasis is still one of the deadly parasitic diseases accounting for significant morbidity and mortality in the Indian subcontinent. As VL is poor man’s disease, the pace of drug development is far away from the requisite perimeter. Most of the marketed antileishmanial drugs are highly toxic and expensive, keeping them out of the grasp of rural India^19, 20^. Widespread drug resistance is another difficult problem to tackle the disease^21^. In this context, development of antileishmanial molecule from our vast natural sources is worthwhile.

A carbazole alkaloid, mahanine, has already been established as a potent anticancer molecule against different types of cancer cells both *in vitro* and *in vivo* with minimal toxicity to normal cells^17^. The main achievement of the present study is to introduce this molecule which exhibited for the first time the anti-proliferative effect on drug sensitive and resistance virulent *L. donovani* promastigotes as well as human infected form amastigotes. Here, we demonstrated the underlying involvement of the various apoptotic-related molecules in mahanine-treated promastigotes through ROS production. We have also shown that mahanine interact with antioxidant enzymes which are involved in ROS generation in *L*. *donovani* through *in-silico* approach. The accumulated ROS further decreased the level of one such antioxidant enzyme (APX). Additionally, mahanine activated the effector molecules (ROS and NO) and also altered immune regulation in infected macrophages for successful clearance of parasite. Taken together, we have for the first time demonstrated the effectiveness of a nontoxic novel herbal molecule as an antileishmanial agent conclusively confirmed by both *in vitro* and *in vivo* experiments.

Apoptosis is the predominant form of program cell death in *Leishmania* ^22^. Here we report that mahanine is inducing a few hallmark of apoptotic events such as membrane flipping, cell cycle arrest and DNA degradation in *L. donovani* parasites. In both prokaryotic and eukaryotic organisms, mitochondria play a pivotal role in ROS generation which could induce cellular redox imbalance and ultimately leads to apoptosis^23^. ROS initiates at the early phase of apoptosis, followed by thiol depletion and cell death^24^. Mahanine exhibited the mitochondrial depolarization which can also lead to the ROS generation in infective stage of the parasite. It induced a significant amount of ROS even at early time point leading to the cell death. The reversal of mahanine-induced apoptosis by glutathione precursor, NAC established the critical role of ROS in this event.

As *Leishmania* is an intracellular form of parasite and amastigote is the infective form in the mammalian host, so the efficacy of mahanine was further tested against amastigotes. It showed a dose-dependent reduction in the intracellular amastigotes. To establish a successful infection, *L. donovani* induces immunosuppressive effect which could lead to deactivation of macrophages effector function. Therefore, an effective antileishmanial response should generate a dominant Th1 response^25^. Mahanine exhibited a dose-dependent increase in IFNγ level in infected macrophages which are corroborated with the gradual decrease in IL-4 and IL-10 associated with immunosuppression suggesting its antileishmanial capacity.

For further validation, we have also tested the status of a major signaling pathway regulating the cytokine secretion. IL-12 can mediate Th1 response leading to IFNγ secretion during *Leishmania* infection^26^. STAT1 and STAT4 being transcription factors of IFNγ and IL-12 respectively are well correlated with enhanced IFNγ levels in mahanine-treated infected macrophages. In contrast, *L. donovani* induces STAT3 expression during infection, which is the inducer of a major immune suppressive cytokine (IL-10)^27^. Downregulation of p-STAT3 in mahanine-treated infected macrophages is further confirming the reversal of immunosuppressive condition confirming critical role of mahanine in the modulation of host’s cytokine response in clearance of the parasite.

IFNγ secretion has a dominant effect on NO production^28^. This was corroborated by the increased nitrite generation by mahanine in the infected macrophages. ROS generation in the host cell is either through a by-product of the cellular metabolism of oxygen or disruption of mitochondrial electron transport chain. Our compound has earlier been proved to be a potent ROS generator through inhibition of complex III of ETC in cancer cells^12^. Mahanine also produced a significant amount of ROS within 1 hr in parasite infected macrophages.

The parasite also can modulate the production of mitochondrial ROS in the macrophages by strong upregulation of a mitochondrial inner protein UCP2, a negative regulator^5^. Induction of this molecule upregulates the phosphatase activity of macrophage which in turn suppresses p38 and ERK1/2^29, 30^. Here we have demonstrated the mahanine induced decrease in UCP2 molecule in the infected macrophages which are further linked to reduced SHP-1 level. Upregulation of p38 and ERK1/2 indicated the enhanced probability of induction of inflammatory response which could be correlated with Th1 type cytokine response and augmented effector functions for clearance of parasites.

*Leishmania* encounters various oxidative stresses in the host cells thus they express several antioxidant enzyme cascades for their survival in the host^7^. Due to the absence of selenium-dependent glutathione peroxidase, tryparedoxin is one of the major enzymes reported to be a virulent protein in *Leishmania* to neutralize oxidative insult^31^. Therefore this may be considered as a potent drug target^32^. Ascorbate peroxidase in another major antioxidant enzyme reported to function during oxidative burst for parasite survival^33^. The parasite also utilizes superoxide dismutase and heat shock proteins for as their defence arsenal^34, 35^.

Mahanine being a prooxidant agent induces ROS and ROS-mediated cell death both in virulent promastigotes and amastigotes, therefore it was worthwhile to test whether it can target anti-oxidant enzymes of the parasite. Computational approach has provided several methods for protein structure prediction and characterization of binding sites, studying the dynamic nature of drug targets and affinity of the protein-ligand complex^36^. Therefore, as a preliminary study, we attempted to determine the strength of binding of mahanine to these enzymes and their preferred orientations in the binding pockets through *in-silico* approach. Currently, we are in a process of in-depth study to validate such computational prediction to compare the involvement of all the related antioxidant enzymes of the parasite in their clearance. Such studies are ongoing.

The therapeutic efficacy of mahanine was successfully corroborated in acute murine models of experimental visceral leishmaniasis. However, there is a limitation for using Balb/c mice due to the differences in clinicopathological features than the active human disease^37^. Hamsters are the good animal model that mimics human infection; however, non-availability of immunological tools restricts its use. As the longer duration of infection in mice induces a cellular response that inhibits parasite growth, we have used 15 days model of infection which provide all the immunological features similar to human. Oral feeding of mahanine for only five consecutive days showed almost complete remission of spleen parasite burden indicating the potency of this molecule. *In vivo* mahanine treatment also associated with strong induction antileishmanial effector molecules, enhanced IL-12 response and T cell proliferation indicating the boost in cell-mediated immunity. The nontoxic nature of this prooxidant molecule might give an additional advantage over synthetic drugs due to its capacity to modulate host’s immune response.

Most of the currents used antileishmanial drugs mainly act through directly targeting the parasite without rejuvenating the host immune response and showed significant toxicity in the tissues^1, 38^. Although miltefosine has immunomodulatory activity, it is associated with acute toxicity like teratogenicity and abortifacient nature with a long median half-life^9^. More importantly, it is very expensive and therefore beyond the reach of most of the patients together with emerging drug resistance. As herbal compounds show a great deal of promise as chemotherapeutic agents against a variety of diseases, we propose mahanine as an alternative inexpensive source since it is nontoxic to normal cells and different organs with no loss of body weight in experimental animals^17^. Thus mahanine might have extra advantages of being nontoxic, inexpensive and isolated from an abundantly available plant.

Taken together our in-depth study conclusively ascertains the function of mahanine on both drug sensitive and drug resistant virulent parasites and has the potential to clear parasite burden *in vivo*. Therefore, we propose the introduction of this nontoxic herbal molecule as a potential antileishmanial agent. There is also the opportunity to use this herbal remedy as a combinational chemotherapeutic agent along with traditional antileishmanial drugs.

## Materials and Methods

### Reagents and mice

DMSO and Giemsa stain were purchased from Sigma (MO, USA); MTS-PMS assay kit was from Promega (WI, USA); Apoptotic DNA Ladder Kit was from Roche (Basel, Switzerland); JC1 dye was from ThermoFisher Scientific (OR, USA), H_2_DCFDA was from Molecular probes (OR, USA); Annexin V-PI assay kit, BDOpt EIA^TM^ELISA set, Superscript first stand synthesis system was from Invitrogen (CA, USA); Cell Cycle Test Plus DNA reagent kit, 7-AAD and anti-SHP1 antibody were purchased from BD Pharmingen and BD Biosciences (CA, USA); UCP2 antibody was from SantaCruz Biotechnology (CA, USA). All other antibodies were purchased from Cell Signaling Technologies (USA). Mahanine was dissolved in 100% ethanol to make 5000 μM for *in vitro* studies and dissolved in >5% DMSO for *in vivo* studies.

Inbred Balb/c mice (20–22 g) were housed in Institute Animal Facilities in CSIR-CDRI and fed a standard diet. All the animal-related experiments were performed in accordance with the National Regulatory Guidelines issued by Committee for the Purpose of Control And Supervision of Experiments on Animals (CPCSEA), Ministry of Environment and Forest, Govt. of India and used for experiments with prior approval from Institutional Animal Ethical Committee (IAEC)(IAEC approval no: IAEC/2015/42D).

### Purification and characterization of mahanine

Mahanine, a carbazole alkaloid was purified from fresh leaves of an Indian medicinal plant, *Murraya koenigii* easily available throughout India. The purity and the structure of the isolated compound were established by different approaches like HPLC, LC-MS, 1[H] and13[C] NMR analysis as stated earlier^10, 17^ (Fig. 1a).

### Parasite and cell culture

*L. donovani* sodium antimony gluconate (SAG) sensitive strain AG83 (MHOM/IN/1983/AG83) originally isolated from an Indian patient with Kala-azar (ATCC® PRA413™) and SAG resistant GE1 (MHOM/IN/90/GE1) were maintained in M-199 medium supplemented with 10% heat-inactivated fetal calf serum (FCS, v/v) and gentamycin sulphate (200 µg/ml) at 22 °C^39^. To ensure the virulence, parasites were routinely passage through hamster. Murine macrophage cell line (J774A.1) was cultured in IMDM supplemented with 10% heat-inactivated FCS and antibiotic antimycotic solutions at 37 °C with 5% CO_2_.

### Promastigote viability assay

Log phase *L. donovani* AG83 promastigotes (0.5 × 10^6^/ml) were plated in 96 well (200 µl/well) plate and incubated with a graded dose of mahanine (0–50 µM) for 24 and 48 hr respectively at 22 °C. MTS (2.0 mg/ml) and PMS (0.92 mg/ml) were added (20 µl/well) in 5:1 ratio and further incubated for 3 hr at 37 °C. A number of formazan crystals formed were quantified by measuring the intensity of colour at 492 nm in an ELISA reader (Thermo Scientific), indicating the number of viable cells^24^. Control cells were exposed to the highest amount of the vehicle (0.15% absolute ethanol).

Promastigotes (2 × 10^6^/ml) were incubated with mahanine (0–30 µM) for 24 hr at 22 °C. 7-AAD was added and further incubated in dark at 22 °C for 30 min according to the manufacturer’s instructions. Cells were acquired in a flow cytometer (BD FACSCalibur, BD Biosciences) by analyzing at least 1 × 10^4^ cells with CellQuestPro software (BD FACSCalibur)^40^.

### Annexin V-PI positivity

Mahanine-induced apoptosis was determined by annexinV-PI positivity as stated earlier with some modifications^41^. Log phase promastigotes (2 × 10^6^/ml) were incubated with mahanine (0–10 µM) for 24 hr at 22 °C. Cells were centrifuged, washed with PBS and suspended in annexin V binding buffer. Annexin V and PI then added according to the manufacturer’s instruction and incubated for 30 min in dark at 25 °C Acquisition was done in FACS and analyzed by CellQuestPro software.

### DNA degradation assay

Mid-log phase promastigotes (1 × 10^7^) were treated with mahanine (0–30 µM) for 24 hr and total DNA was extracted from treated and untreated cells using apoptotic DNA extraction kit as per manufacturer’s instructions, electrophoresed in 1% agarose gel and stained with ethidium bromide (0.50 µg/ml) in Tris-boric acid-EDTA (TBE) buffer. The image was visualized and photographed in Bio-Rad Gel Documentation system^24^.

### Cell cycle analysis

Mahanine-induced cell cycle arrest was determined by cell cycle kit as stated earlier with some modifications^12^. Exponential phase parasites (2 × 10^6^) were treated with mahanine (0–15 µM) for 24 hr at 22 °C. They were harvested and processed using Cell Cycle Test Plus kit. At least 20,000 cells were acquired and the percentage of cells present in different phases of the cell cycle was determined by FACS. Data analyzed by CellQuestPro software.

### Analysis of mitochondrial membrane potential

Mitochondrial membrane potential was measured using a cell permeable JC1 dye with some modifications^41^. Briefly, log phase promastigotes (2 × 10^6^/ml) were incubated with mahanine (0–30 µM) for 24 hr at 22 °C. The cells were suspended in phosphate-buffered saline (PBS) and stained with the JC1 dye (2 µM/tube) for 10 min at 37 °C analyzed by flow cytometry.

### Measurement of intracellular reactive oxygen species (ROS) in promastigotes

Mahanine-induced intracellular ROS generation was measured by 2′7′- dichlorodihydro fluorescein diacetate acetyl ester (H_2_DCFDA) staining with some modifications^24^. For time kinetics, mid-log phase promastigotes (2 × 10^6^) were preloaded with H_2_DCFDA (50 µM) in PBS for 45 min at 37 °C in dark. Cells were washed and suspended in M199 medium supplemented with FCS. They were treated with the IC_90_ dose of mahanine (25 µM) for 0–1 hr at 22 °C in dark. For dose kinetics study, promastigotes were processed similarly and incubated with mahanine (0–30 µM) for 45 min in dark. The inhibition of ROS generation was shown at the highest dose of mahanine by pretreatment of cells with NAC (2.5 mM) for 30 min. Cells were washed, suspended in PBS. Intracellular H_2_O_2_ was determined using flow cytometry by analyzing 10,000 cells.

### Analysis of ROS-dependent death of promastigotes

Exponential phase promastigotes were suspended in M199 medium. One set of cells were pretreated with NAC (2.5 mM) for 30 min and washed. NAC-treated and untreated promastigotes were plated (2 × 10^6^/well) in a six-well plate and incubated with mahanine (0–15 µM) for 24 hr at 22 °C. Cells were spin down, washed and suspended in PBS. PI (5.0 µg/ml) was added to 5.0 µl and incubated for 30 min in dark. Cells were washed, resuspended in PBS and acquired in FACS. Data was analyzed in CellQuestPro software^42^.

### Effect of mahanine on amastigote in infected macrophage

Microscopic analysis of the effect of mahanine in intracellular amastigote number was carried out as described earlier^43^. J774A.1 (2 × 10^4^) was seeded in glass coverslip and incubated for 48 hr at 37 °C incubator containing 5% CO_2_ for adherence. Cells were infected with stationary phase AG83 promastigotes at 1:10 (macrophage: parasite) ratio in for 4 hr at 37 °C. Non phagocytosed parasites were removed, washed and infected J774A.1 cells were kept another 20 hr for multiplication of the parasite. Mahanine (0–20 µM) was added and incubated for another 24 hr. The medium was removed; coverslip was air-dried, fixed in methanol and Giemsa stained. The intracellular amastigote (100 macrophages/cover slip) was counted using a Zeiss microscope at 100X resolution in oil immersion.

### Measurement of secreted cytokines in infected macrophages

J774A.1 cells were infected and treated with mahanine as described in the earlier paragraph in presence of LPS (2.5 µg/ml) for secretion of Th2 or PMA (25 ng/ml)-ionomycin (1.0 µg/ml) for Th1 cytokines overnight. Secreted cytokines (IFNγ, IL-4, and IL-10) was measured in the cell-free suspension by ELISA kit and the colour intensity was measured at 450 nm in ELISA reader^43^.

### Western blot analysis

J774A.1 cells (1 × 10^6^) were infected with promastigotes at 1: 10 at as stated earlier. Both uninfected and infected cells were incubated with mahanine (0–15 µM) for 24 hr. The cell lysate was prepared and an equal amount of protein was separated in an SDS-PAGE (10%) and transferred to nitrocellulose membrane. The membrane was blocked with tris buffer saline (TBS)-BSA (2%) and incubated with the specific primary antibody (1:1000 dilutions) overnight at 4 °C followed by adding species specific HRP-conjugated secondary antibody (1:1000 dilutions). The signal was detected by West-pico ECL system (Pierce, Thermo Scientific, USA) as described earlier^15^.

*L. donovani* promastigotes (2 × 10^7^) were treated with mahanine (0–20 µM) for 24 hr at 22 °C. The cell lysate was prepared and separated in 12% SDS-PAGE. Western blot was performed as stated above.

### ROS generation by infected macrophages/splenocytes

Mahanine-induced ROS generation by infected macrophages was measured as stated previously with some modifications^39^. Briefly, J774A.1 cells (1 × 10^6^ cells/well) were infected with stationary phase promastigotes in 1:10 ratio for 4 hr in 37 °C. After the infection, non-ingested parasites were removed and infection was allowed for 20 hr in 37 °C. Then a graded dose of mahanine (0–15 µM) was added to infected and uninfected macrophages and further incubated for 24 hr at 37 °C. Macrophages were pellet down, washed and stained with H_2_DCFDA (10 µM) for 30 min at 37 °C and processed similarly.

For in vivo, splenocytes isolated from all the experimental groups of mice were plated (1 × 10^6^ cells/well) and incubated at 37 °C/5% CO_2_ for 72 hr in presence of SLA (soluble *Leishmania* antigen, 50 µg/ml). Splenocytes were then incubated with 10 μM H_2_DCFDA at 37 °C for 20 min and then analysed on fluorescence-activated cell sorter (FACS) Calibur flow cytometer (Becton Dickinson, USA) using a 530-nm filter^44^.

### Nitric oxide (NO) measurement in the culture supernatant of infected macrophages/ splenocytes

NO was measured by Griess reaction^39^. Briefly, J774A.1 cells (1 × 10^6^/well) plated in a six-well plate and infected with stationary phase promastigotes at 1:10 ratio for 4 hr at 37 °C. After the infection, unbound parasites were washed out and infection was allowed for 20 hr at 37 °C. Mahanine (0-15 µM) was added to the wells and incubated for 24 hr. Culture supernatants were used to measure accumulated nitrite in the ELISA reader (Thermo Scientific) at 540 nm. Sodium nitrite (NaNO_2_) diluted in culture medium was used as a standard.

For *in vivo*, splenocytes from different experimental groups of Balb/c mice was prepared by Ficoll density gradient centrifugation and incubated for 3 days in 5% CO_2_ incubator at 37 °C in presence of soluble *Leishmania* antigen (SLA). NO generation was quantified by measuring the accumulation of nitrite in culture supernatants by Griess reagent^45^.

### Molecular modeling studies

#### Target sequence and template identification

The amino acid sequences of all the antioxidant enzymes of *L. donovani* namely ascorbate peroxidase (H6V7N3_LEIDO), mitochondrial tryparedoxin peroxidise (A4ZZ67_LEIDO), cytoplasmic tryparedoxin peroxidase (A4ZZ66_LEIDO), Hsp83 (HSP83_LEIDO), superoxide dismutases, namely, FeSODA (Q71S86_LEIDO) and FeSODB1 (Q71S88_LEIDO) were obtained from the UniProt database. For the template identification of these antioxidant enzymes for their 3D structure prediction, BLASTp was performed taking the sequence of these proteins by searching against Protein Data Bank (PDB).The selected templates for these antioxidant enzymes are tabulated in Table 1.

#### Homology model building and refinement

The homology models of these enzymes were constructed using Modeller v9.11^46, 47^.For each enzyme, a total of 100 modeled structures were generated, based on the target sequence, target–template alignment information and 3D structure of the template proteins (from PDB). Spatial restrictions such as bond lengths, bond angles, dihedral angles and interactions between non-bonded residues were also considered and finally ranked them by the molecular probability density function (MolPdf) score. Sequential loop refinement was also performed using loop refinement tool after model building. The structure with the lowest MolPdf score was taken and was repeatedly energy minimized to get the lowest energy structure using Discovery Studio (Accelrys).

#### Model validation

The stereo-chemical quality of all the modeled structures of six antioxidant enzymes were evaluated by Ramachandran plot generated by RAMPAGE (http://mordred.bioc.cam.ac.uk/~rapper/rampage.php,^48^, Verify3D^49^ and ERRAT^50^. Finally, the root means square deviation (RMSD) between the backbone of the template and the target was computed using the Struct program from Schrödinger.

#### Identification of binding sites

Using protein preparation wizard of Maestro package version 15.2 (from Schrödinger, LLC), the protonation state of the ionizable residues were fixed by using the Protein Preparation Wizard. In order to identify the binding pocket, the site recognition program SiteMap (Maestro 10.3.014) was run on all these modeled structures of six antioxidant enzymes^51^. This program calculates two types of scores for each site, namely SiteScore and Druggability score (Dscore). The putative binding pockets were selected on the basis of criteria that the Dscore was higher than 1.0.

#### Docking studies

The structure of mahanine was retrieved from PubChem, gastegier charges were added and typed with the cff91 force field. The structure was optimized with repeated energy minimization and molecular dynamics simulations using simulation module of Discovery Studio (Accelrys). Energy minimization was performed alternately with steepest descent and conjugate gradient methods (200 steps each). Molecular dynamics simulation was done for 10,000 steps of 1 fs after 1000 steps of equilibration with a conformation sampling of one in 100 steps at 300 K. The optimized structure was used for docking studies. Molecular docking of mahanine with each of the antioxidant enzymes was performed using Autodock 4.2^52, 53^. Protein structures were prepared by adding hydrogens. Non-polar hydrogens were merged and gastegier charges were assigned. Similarly, gastegier charges computed by Autodock tool were used on each atom of the ligand. AutoTors utility was used to define torsional degrees of freedom for the ligand. Autogrid was used to perform grid map settings. The grid was prepared around the binding sites as predicted by SiteMap program with 88 × 80 × 80 Å cube and the spacing was at 0.375 Å. The lamarckian genetic algorithm was used to perform docking simulation, with an initial population of 150 randomly placed individuals, a maximum number of 2,50,000 energy evaluations, 1,50,000 generations, the mutation rate of 0.02, a cross over rate of 0.8 and an elitism value of 1 were used. 100 docking runs were performed. Pseudo-Solis and Wets algorithm was used for local search method. Finally, the resulting docked conformations were clustered together on the basis of RMSD tolerance of 1.5 Å and represented by a most favourable free energy of binding.

### Genetic expression profiling APX in promastigotes

Log phase *L. donovani* AG83 promastigotes (1 × 10^7^/ml) were treated with mahanine (0–20 µM) for 24 hr in 22 °C. Total RNA was isolated from promastigote by using Lipid Tissue RNA isolation Kit. RNA was transcribed to cDNA and the specific primers for the gene of APX (forward, 5′CTGTACGAGGGTAACAAAGG3′; reverse, 5′ATCAGCTGCATCTTCTCAAC3′) and α-tubulin (forward, 5′CATCTACGACCTCACTCGTC3′; reverse, 5′GACAATTCAGTTCGTGGACT3′) was used for amplification in RT-PCR. The reaction product was separated in 1% agarose gel stained with ethidium bromide and image captured in Bio-Rad Gel Documentation system^54, 55^.

### Efficacy of mahanine in *Leishmania*-infected acute murine model

Female Balb/c mice (4–6 weeks) were infected with stationary phase AG83 promastigotes (1 × 10^7^) suspended in PBS by lateral tail vain. After 15 days post-infection, animals were selected for subsequent experiments^45^.

Three groups of mice, each containing 5 animals was used for treatment with mahanine. Group I mice was fed with PBS only as vehicle control, while group II and III received 20 mg/Kgb.w/day and 40 mg/Kgb.w/day mahanine for 5 consecutive days respectively and sacrificed after 4 days post-feeding. Standard drug miltefosine was used as a reference at 30 mg/Kgb.w/day for 5 consecutive days similarly. Parasite burden was calculated in Leishman-Donovan Unit (LDU), LDU = amastigote per nucleated cell × organ weight in milligram^45^.

### T cell proliferation assay

T cell proliferation assay was performed as described earlier^55^. Briefly, isolated splenocytes (1 × 10^5^/well) from mice were plated 96-well plate and incubated for 3 days at 37 °C/5% CO_2_ for proliferation. Next, 3-(4,5-dimethylthiazol-2-yl)-2,5-diphenyl tetrazolium bromide (MTT, 1.0 mg/ml/well) was added and further incubated for 4 hr. Resultant formazan crystals were dissolved in DMSO and the colour intensity was determined in ELISA reader at 492 nm.

### Real-time PCR analysis of cytokines

Total RNA was isolated from infected and uninfected mice splenocytes by using an RNeasy mini kit as described previously. cDNA was synthesized using RNA (1.0 mg) as a template; real-time PCR analysis was performed by mixing cDNA with SYBR Green PCR Master mix and amplified using primers specific for genes encoding cytokines IL-12 (forward, 5′ TATGTTGTAGAGGTGGACTG 3′; reverse, 5′ TTGTGGCAGGTGTATTGG 3′) and *iNOS* (forward, 5′ CGACGGCACCATCAGAGG 3′; reverse, 5′ AGGATCAGAGGCAGCACATC 3′) in Roche Applied Science light cycler 480.0. Relative quantification of each target genes was normalized to mRNA level of a housekeeping gene (HGPRT-forward, 5′ GATAGATCCACTCCCATAACTG 3′; reverse, 5′ TACCTTCAACAATCAAGACATTC 3′) and expressed as a fold change compared with uninfected control using the comparative cycle threshold (CT) method^45^.

### Statistical analysis

Data represented are the mean ± standard error of the mean for three independent experiments. Data were analyzed by one-way ANOVA using GraphPad Prism software (version 5.1). Student’s t-test was performed to measure the statistical significance of differences between a pair of data sets. Differences in p- values of ≤0.05 between treatment groups were considered significant.

## Electronic supplementary material

Supplementary Info
