# Supplementary material for: Mahanine exerts in vitro and in vivo antileishmanial activity by modulation of redox homeostasis
Source: Sci Rep. 2017 Jun 23;7:4141. doi: 10.1038/s41598-017-03943-y (PMC5482887; doi:10.1038/s41598-017-03943-y)
Supplement: Supplementary file 1 — Supplementary Info [file 41598_2017_3943_MOESM1_ESM.pdf]

Mahanine exerts *in vitro* and *in vivo* antileishmanial activity by modulation of redox homeostasis

Saptarshi Roy[a], Devawati Dutta[a], Eswara M Satyavarapu[a], Pawan K Yadav[b], Chhabinath Mandal[c], Susanta Kar[b], Chitra Mandal[a]*

[a]Cancer Biology and Inflammatory Disorder Division, Council of Scientific and Industrial Research (CSIR)-Indian Institute of Chemical Biology, 4, Raja S.C. Mullick Road, Jadavpur, Kolkata-700032, India

[b]Division of Parasitology, CSIR-Central Drug Research Institute, Lucknow-226001, India

[c]National Institute of Pharmaceutical Education and Research, Kolkata, 4 Raja S. C. Mullick Road, Kolkata 700032, India

Running Title:

Antileishmanial activity and redox imbalance by mahanine

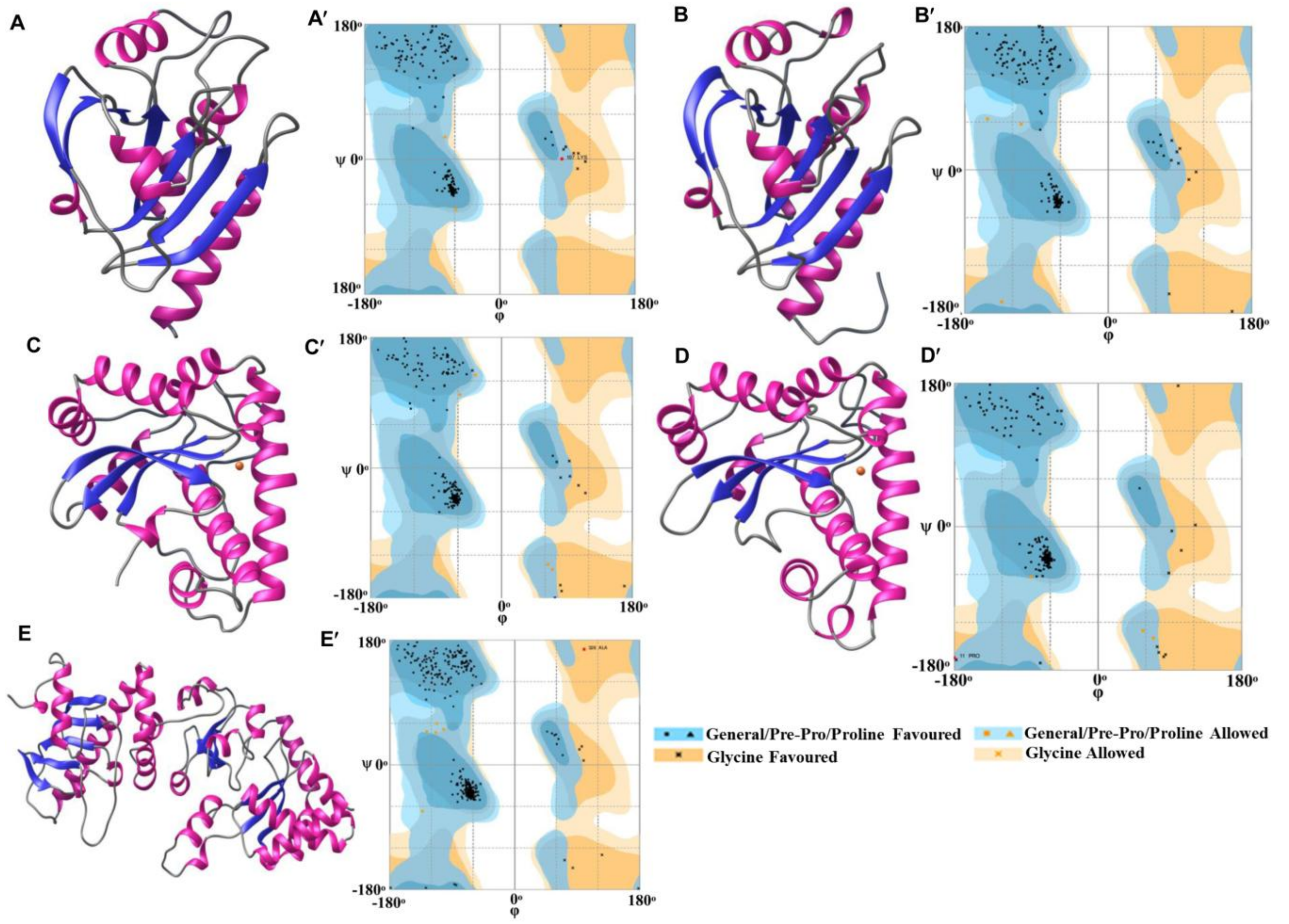

Supplementary Fig S1: Homology modeling of antioxidant enzymes of *L. donovani*

Modeled structures and Ramachandran maps of (A, A´) Tryperedoxin peroxidase (Cytoplasmic), (B, B´) Tryperedoxin peroxidase (Mitochondria), (C, C´) Superoxide dismutase (FESODA), (D, D´) FESODB1 and (E, E´) HSP83.The structure is displayed in ribbon form where colors are represented as (sheets = dark blue, helices = magenta and loop = grey).

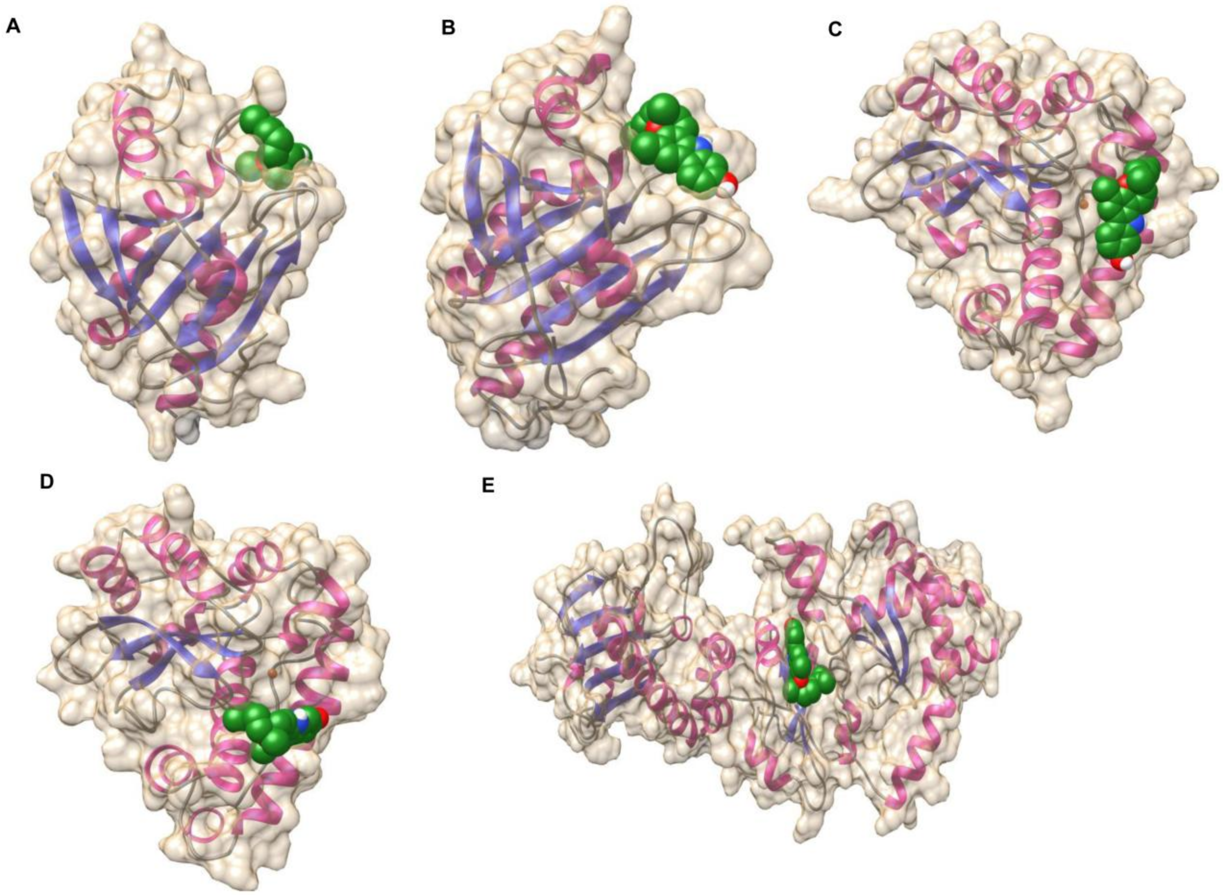

Supplementary Fig S2: Docked complexes of modeled antioxidant enzymes

(A) Tryperedoxin peroxidase (Cytoplasmic), (B) Tryperedoxin peroxidase (Mitochondria), (C) Superoxide dismutase (FESODA), (D) FESODB1 and (E) HSP83 with mahanine. Mahanine is represented in sphere form where C=green, O=red, N= dark blue and H=white. The protein is represented both as a partial Connolly surface in tan colour and ribbon form with helices (magenta) and sheets (dark blue).
